# Outcomes of extensive hemilaminectomy with durotomy on dogs with presumptive progressive myelomalacia: a retrospective study on 34 cases

**DOI:** 10.1186/s12917-020-02690-z

**Published:** 2020-12-07

**Authors:** Ryuji Hirano, Ryota Asahina, Taiyo Hirano, Ayuko Hyakkoku, Rino Miura, Takuya Kunihiro, Yuya Nakamoto

**Affiliations:** 1 Ukyo Animal Hospital, 12-2 Uzumasa-Kyonomichicho, Ukyo-ku, Kyoto, Japan; 2 Kyoto Animal Medical Center, 550-4 Bishamoncho, Nakagyo-ku, Kyoto, Japan; 3 Graduate School of Medicine, Kyoto University, 54 Shogoin-Kawaharacho, Sakyo-ku, Kyoto, Japan; 4 Neuro Vets Animal Neurology Clinic, 550-4 Bishamoncho, Nakagyo-ku, Kyoto, Japan

**Keywords:** Canine, Extensive hemilaminectomy with durotomy, Progressive myelomalacia, Thoracolumbar intervertebral disk herniation

## Abstract

**Background:**

Progressive myelomalacia (PMM) is a fatal complication of progressive ascending and descending necrosis of the spinal cord after acute spinal cord injury. A recent study suggested that extensive hemilaminectomy with durotomy (EHLD) at the intramedullary T2-hyperintense region which performed immediately after magnetic resonance imaging (MRI) improved the survival rate in dogs with presumptive PMM. The objective of this retrospective study was to evaluate the effects of EHLD on halting the progression of PMM in dogs presumptively diagnosed with PMM which had the interval between MRI and surgery.

**Results:**

Thirty-four dogs with presumptive PMM which had undergone EHLD with the delay following MRI examination (range, 0 to 3 days) were included. The cranial side of EHLD was set depending on the delay time after MRI, MRI findings, neurological examination and intraoperative macroscopic appearance. Two weeks after surgery, the perioperative survival rate was 97% (33/34). During follow-up with a median time period of 82.5 weeks (range, 0-290 weeks), the postoperative survival rate was 91% (31/34). At the end of the follow-up period, 31 out of 34 dogs were alive without severe postoperative complications while the remaining 2 dogs died from causes not directly attributable to the surgery. There was no improvement in the pelvic limb function of all dogs.

**Conclusions:**

EHLD appears to be effective in halting the progression of presumptive PMM and preventing morbidity even in dogs which had the interval between MRI and EHLD. Our algorithm of determining the range of EHLD may enable to set the appropriate ranges of EHLD in the cases which develop signs consistent with PMM after MRI examination.

## Background

Progressive myelomalacia (PMM) is a fatal disease characterized by progressive ascending and descending necrosis of the spinal cord after acute spinal cord injury [1, 2]. PMM is frequently observed following thoracolumbar intervertebral disk herniation (IVDH) with no deep pain perception (NDPP) in the pelvic limbs [1, 3]. The prevalence of PMM with IVDH has been estimated at 2%, ranging from 0–15% depending on clinical grade [3]. In paraplegic dogs with NDPP, its prevalence ranges from 9 to 25% [2, 4–8].

The initial clinical signs of PMM are lower motor neuron signs in the pelvic limbs and cranial advancement of the caudal border of the cutaneous trunci muscle (CTM) reflex. CTM originates in the caudal gluteal region and runs cranially and ventrally to insert into the axillary region, where it connects with the latissimus dorsi muscle and the deep pectoral muscle [9]. The CTM reflex is commonly used to localize thoracolumbar spinal lesions and its caudal movement is considered a reliable predictor of recovery in dogs affected by different pathologies including acute thoracolumbar myelopathies [10, 11].

Following the onset of PMM, loss of anal and abdominal tone, plegia of the thoracic limbs and respiratory muscles develop. Most dogs are euthanized before respiratory failure result in their deaths [1, 2]. The diagnosis is suspected by the course of clinical signs and corresponding magnetic resonance imaging (MRI) findings before confirmation with histology demonstrating necrosis of the spinal cord. An intramedullary hyperintense signal longer than 6 times the length of L2 vertebral body on T2-weighted images on MRI is suggestive of PMM [12]. An extensive intramedullary hyperintensity has also been reported in dogs with IVDH that did not develop myelomalacia and with other myelopathies [1, 13, 14]. Since some dogs develop signs consistent with PMM after MRI examination, the absence of intramedullary T2 hyperintensity does not preclude the development of PMM [1].

Although the pathophysiological mechanism of PMM is poorly understood, it involves primary mechanical damage due to the spinal cord concussion and contusion caused by the disk herniation, and secondary damage caused by ischemia, edema, electrolyte shifts, oxidative stress, inflammation, and apoptosis [15–17]. A recent study showed elevated intramedullary pressure associated with hemorrhage further leads to the progression of spinal cord destruction [15].

Both durotomy and extended thoracolumbar durotomy were previously shown to improve the rate of regaining ambulation in dogs with IVDH and NDPP in the pelvic limbs [18, 19]. The improved outcome after durotomy or extensive durotomy seemed to result from physical decompression of the spinal cord [18, 19]. Additionally, performing durotomy seemed to prevent the development of PMM in dogs with IVDH and NDPP in the pelvic limbs [19]. Another retrospective study also reported that prompt surgical decompression and treatment with corticosteroids were associated with lower odds of developing PMM in dogs with IVDH and NDPP [20].

Since several studies reported that elevated intramedullary pressure is involved in the pathophysiology of PMM [15, 21, 22], spinal decompression by surgery may be effective against the progression of PMM. A recent study suggested that the extensive hemilaminectomy with durotomy (EHLD) at the intramedullary T2-hyperintense region on MRI improved the survival rate of dogs with presumptive PMM compared with dogs underwent standard hemilaminectomy [23]. In the study, EHLD was performed immediately after they underwent MRI under sustained anesthesia [23]. However, it is unclear that EHLD can be applied in dogs with a delay after MRI because the myelomalacia progress over time in dogs with PMM. The purpose of the present study was to investigate the effectiveness and appropriate ranges of EHLD against the progression of PMM with a time lag after MRI.

## Results

### Study population

Thirty-four dogs with presumptive PMM underwent EHLD (Table 1). Median age was 5.1 years (range, 2.8–13.8 years) at the time of surgery. There were 21 males (9 castrated) and 13 females (7 spayed). Breeds were 23 Miniature Dachshunds, 8 Toy Poodles, 2 French Bulldogs and 1 Chihuahua.

**Table 1:** Baseline characteristics, operative details, and follow up of dogs which underwent EHLD for presumptive PMM

No.	Breed	Age (years)	Sex	CP of thoracic limbs	Site of IVDH	Range of EHLD	Interval between onset and EHLD (days)	Interval between MRI and EHLD (days)	Preoperative Drugs	Survival outcome	Follow-up (weeks)
1	Toy Poodle	3.9	SF	+ 2	L3-4	T9-L4	1	0	Prednisone	Alive	290
2	Miniature Dachshund	3.3	SF	+ 2	T12-13	T4-13	4	0	None	Alive	200
3	Miniature Dachshund	8.6	M	+ 2	T13-L1	T9-L1	3	0	None	Alive	165
4	Miniature Dachshund	4.3	F	0	L1-2	T1-L2	5	0	None	Alive	164
5	French Bulldog	6.9	SF	+ 1	L1-2	T1-L2	5	1	None	Alive	158
6	Miniature Dachshund	12.8	M	+ 2	T13-L1	T1-L1	3	0	Prednisone	Dead (Food aspiration, day 54)	7
7	Miniature Dachshund	13.8	SF	+ 2	T12-13	T3-13	4	1	None	Alive	137
8	Toy Poodle	3.3	F	+ 1	T11-12	C7-T12	5	0	Prednisone	Alive	130
9	Toy Poodle	7.1	CM	+ 2	L1-2	T3-L2	3	1	Prednisone	Alive	127
10	Miniature Dachshund	3.9	M	+ 2	T12-13	T4-13	1	0	Unknown	Alive	125
11	Miniature Dachshund	4.1	M	+ 2	T12-13	T2-13	3	1	Unknown	Alive	125
12	Miniature Dachshund	5.4	CM	+ 2	T13-L1	T5-L1	6	0	Unknown	Alive	125
13	Miniature Dachshund	3.8	F	+ 2	L1-2	T2-L2	3	1	Unknown	Alive	120
14	Toy Poodle	10.5	SF	+ 2	T13-L1	T3-L1	7	0	Unknown	Dead (Uremia, day 56)	8
15	Miniature Dachshund	3.3	M	+ 2	T13-L1	T7-L1	2	0	None	Alive	112
16	Miniature Dachshund	6	CM	+ 2	T13-L1	T5-L1	2	0	None	Alive	111
17	Miniature Dachshund	4.4	F	+ 1	L3-4	T1-L4	4	3	Prednisone	Alive	109
18	Miniature Dachshund	7.4	F	+ 1	T11-12	T1-12	3	1	None	Alive	100
19	Miniature Dachshund	4.8	CM	0	L3-4	C7-L4	4	0	None	Alive	91
20	Toy Poodle	6.6	M	+ 2	L3-4	T10-L4	3	1	Prednisone	Alive	74
21	Miniature Dachshund	4.5	CM	+ 2	T13-L1, L2-3	T3-L4	3	1	Prednisone	Alive	65
22	Toy Poodle	2.8	CM	+ 2	T13-L1	T3-L1	5	1	Unknown	Alive	64
23	Miniature Dachshund	8.7	M	0	T11-12, T13-L1	T1-L1	3	0	Unknown	Dead (Respiratory failure, day3)	0
24	Miniature Dachshund	6	F	+ 1	T9-13 (multiple)	T1-T13	4	2	Unknown	Alive	59
25	Toy Poodle	6.1	M	+ 2	L5-6	T13-L6	1	0	Unknown	Alive	59
26	French Bulldog	4	CM	+ 2	L5-6	T8-L5	3	0	None	Alive	58
27	Miniature Dachshund	10	M	+ 1	L3-4	C7-L4	4	3	Unknown	Alive	48
28	Chihuahua	4	M	+ 2	L4-5	T5-L5	3	0	Unknown	Alive	48
29	Miniature Dachshund	5.3	CM	+ 2	T10-11	T3-T11	3	1	Prednisone	Alive	38
30	Miniature Dachshund	4.4	M	+ 2	L2-3	T3-L3	3	0	Unknown	Alive	27
31	Miniature Dachshund	2.8	SF	+ 2	T12-13	C7-T13	9	0	Prednisone	Alive	25
32	Toy Poodle	2.6	CM	+ 1	L3-4	C7-L4	5	1	Unknown	Alive	23
33	Miniature Dachshund	11	SF	+ 2	T11-12	T2-T12	4	0	Prednisone	Alive	22
34	Miniature Dachshund	5.4	M	+ 1	T13-L1	C7-L1	7	1	None	Alive	21

*CM* castrated male; *CP* Conscious proprioception; *EHLD* extensive hemilaminectomy with durotomy; *F* female; *IVDH* thoracolumbar intervertebral disk herniation; *M* male; *MRI* magnetic resonance imaging; *PMM* progressive myelomalacia; *SF* spayed female.

### History and clinical signs

Clinical signs before surgery included paraplegia with NDPP (all dogs), plegia of the thoracic limbs (3 dogs) and paresis of the thoracic limbs (8 dogs). Mean duration from onset of non-ambulatory obtained through owner interviews to surgery was 3.8 days (range, 1 day to 9 days). Before referral for surgery, prednisone had been administered to 10 dogs. Medical treatment before surgery of 13 dogs was unknown.

### MRI findings

Distribution of T-L IVDH site was T10-11 (1), T11-12 (3), T12-13 (5), T13-L1 (8), L1-2 (4), L2-3 (1), L3-4 (6), L4-5 (1), L5-6 (2) and multiple (3). Mean length of intramedullary hyperintense region was 13.9 (range, 6.7 to 21.7) times of the L2 vertebral body. No dogs underwent EHLD immediately after MRI. Mean duration from MRI to surgery was < 24 hours (19), 24–48 hours (12), 48–72 hours (1) and 72–96 hours (2).

### Surgical details and histological findings

**Fig. 1:**
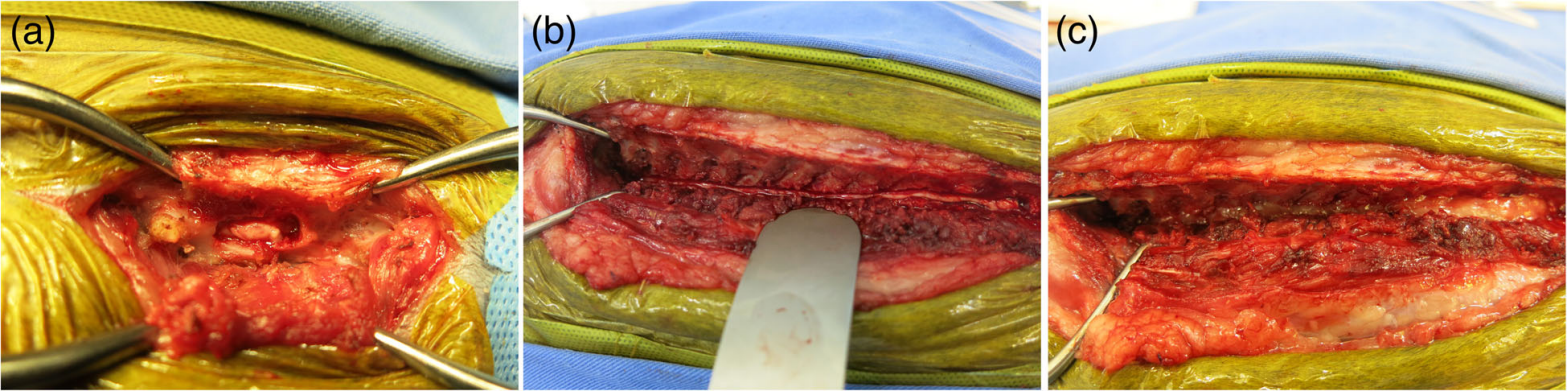
Intraoperative image of EHLD for a dog with presumptive PMM (dog No.14). During hemilaminectomy and durotomy at the IVDH site, softening and liquefaction of spinal cord can be seen (**a**). Extensive hemilaminectomy with durotomy was performed (**b**). Applying a thin layer of subcutaneous fat graft to the hemilaminectomy site before wound closure (c)

EHLD was performed in all dogs (Fig. 1). Extruded intervertebral disk material was present in all dogs and their spinal cords exhibited extensive gross softening and liquefaction macroscopically. In all dogs, a confirmed histological diagnosis of myelomalacia was made through biopsy in the softened spinal cord at the site of extruded intervertebral disc. The mean vertebral body-length window of the EHLD was 12 (range, 5 to 20). The cranial side of the EHLD was C7 (6), T1 (7), T2 (3), T3 (7), T4 (2), T5 (3), T7 (1), T8 (1), T9 (2), T10 (1), T13 (1). There were no intraoperative complications.

### Perioperative outcome

Two weeks after surgery, the perioperative survival rate was 97% (33/34, Table 2). One dog (No.23) which had tetraplegia died 3 days after surgery with respiratory failure (Tables 1 and 2). The dog belonged to the subgroup of cases which underwent EHLD within 24 hours after MRI examination (Table 2). Perioperatively, 15 dogs (44%) experienced fever (range, 39.1–40.2℃) between 1 day and 12 days after surgery. They were administered chloramphenicol and intravenous fluid infusion and recovered within a few days.

**Table 2:** Survival outcome of dogs which underwent EHLD for presumptive PMM

		CP of thoracic limbs			Interval between MRI and EHLD	
	Total (*n* = 34)	Absent (*n* = 3)	Impaired (*n* = 8)	Intact (*n* = 23)	< 24 hours (*n* = 19 )	> 24 hours (*n* = 15 )
Perioperative (< 2-week) survival	33 (97%)	2 (67%)	8 (100%)	23 (100%)	18 (95%)	15 (100%)
Postoperative (> 2-week; range, 9-270 week) survival	31 (91%)	2 (67%)	8 (100%)	21 (91%)	16 (84%)	15 (100%)

*CP* Conscious proprioception; *EHLD* extensive hemilaminectomy with durotomy; *MRI* magnetic resonance imaging; *PMM* progressive myelomalacia.

#### Postoperative outcome and follow-up

**Fig. 2:**
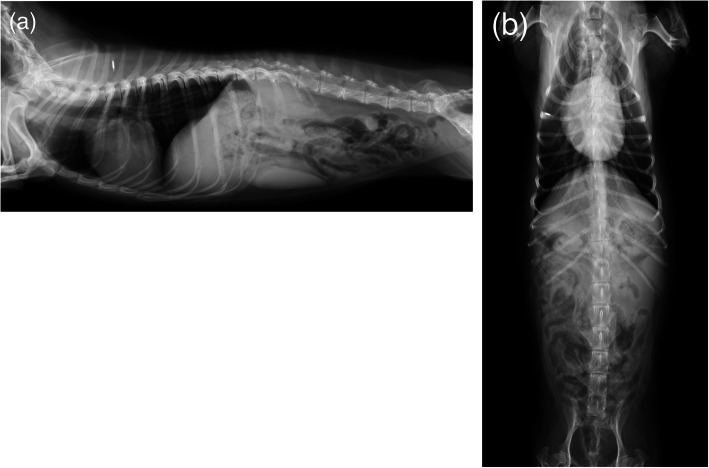
Right lateral (**a**) and dorsoventral (**b**) radiographic images of the thoracolumbar vertebral column at re-examination 8 weeks after surgery, showing no radiological evidence of spinal luxation or subluxation (dog No.31)

No dogs were lost to follow-up. During follow-up with a median time period of 82.5 weeks (range, 0-290 weeks), the postoperative survival rate was 91% (31/34, Table 2). Of the two dogs which died, one died 54 days after surgery (No. 6) from aspiration pneumonia, at home. The other dog died 56 days after surgery (No. 14) from renal failure secondary to pyelonephritis due to an ascending urinary tract infection at another hospital. Both dogs belonged to the subgroup of cases which had intact CP of the thoracic limbs, and the intervals were less than 24 hours between MRI and surgery (Table 2). All remaining dogs were alive during follow-up period. Although they had sporadic urinary tract infections, no serious postoperative complications occurred in this study. None of the dogs underwent spinal stabilization. No dogs had radiological evidence of spinal luxation or subluxation 2 weeks and 8 weeks after surgery (Fig. 2). In addition, 12 dogs were had radiographic evaluation at a median time period of 75.5 weeks after surgery (range, 13 to 204). Similarly, these dogs did not have radiological evidence of postoperative complications. None of the dogs experienced clinical improvement of pelvic limb function. In contrast, neurological function of the thoracic limbs was improved within 2 months after surgery in all 8 dogs with paresis of the forelimbs although it was not observed in the dogs with tetraplegia during follow-up period.

## Discussion

This study demonstrates EHLD as a potentially life-saving treatment for dogs with presumptive PMM, similar to a previous study [23]. Additionally, the present study suggests that EHLD can apply to the cases with the delay following MRI examination. Typically, dogs diagnosed with PMM followed a fatal course [1, 3, 12]. According to the literature, the majority of dogs with presumptive and confirmed PMM are euthanized within 4 days following onset although delayed progression to euthanasia may take as long as 2 weeks [1]. The perioperative survival rate in this study at 2 weeks after surgery was 97% (31/34), indicating high survival rate. It has been reported that elevated intramedullary pressure is involved in the pathophysiology of PMM [15, 21, 22]. More specifically, in PMM, spinal cord debris together with cerebrospinal fluid accumulating in the central canal are transported cranially and caudally due to the increased intramedullary pressure causing a broadening of the tissue necrosis and hemorrhagic lysis of the spinal cord segments. The dura mater plays an essential role in the function of the spinal cord. Representing the strongest structure of the meninges, it sustains the flow and pressure of the cerebral spinal fluid due to its stiffness in circumferential direction [22, 24, 25]. In an experimental edema model using the spinal cords of human cadavers, the pia mater was also showed to be involved in increasing intramedullary pressure [26]. However, in the previous studies on human medicine, the physical therapy of incising the dura seems to achieve sufficient decompression effect on reducing intramedullary pressure [27, 28]. Therefore, we hypothesized that extensive incision of the dura mater and hemilaminectomy in dogs with PMM could reduce intramedullary pressure. It was already observed that decompression with durotomy is possibly an effective treatment for recovering ambulation in severe acute IVDH [18, 19]. These studies showed that durotomy in combination with decompression could potentially prevent the development of PMM in dogs with IVHD with NDPP of the pelvic limbs [18, 19]. The authors discussed that a durotomy might not prevent further progression in dogs advanced PMM [19]. However, a recent study [23] and the present study indicate that spinal decompression with EHLD halts further progression of PMM even for cases with a presumptive diagnosis of advanced PMM.

The extend of hemilaminectomy and durotomy should be carefully considered. A previous study showed that the durotomy extending for four vertebral lengths improved functional outcome in dogs with severe spinal cord injury after acute IVDH [18]. Selection of this length was based on previous studies on the length of spinal cord swelling estimated from imaging findings [14, 29]. In general, hyperintensity in T2-weighted images on MRI represents the liquid component. In dogs with presumptive and confirmed PMM, the T2-weighted images on MRI of the spinal cord frequently shows hyperintensity due to irreversible progressive necrosis of the parenchyma with or without intradural hemorrhage [12]. Thus, EHLD should be performed with the hyperintense region on T2-weighted image taken as the affected portion of PMM. In a recent study, EHLD at the site of T2-hyperintense region was immediately performed after MRI examination in dogs with presumptive PMM following IVDH [23]. All dogs enrolled in our study already underwent MRI at other referral centres before surgery, and the delay to surgery prompted hemilaminectomies and durotomies even longer than T2-hyperintense lengths on MRI. In cases operated on the day after MRI, the cranial side of the EHLD range was set to 1 additional vertebral body to the cranial side of the T2-weighted hyperintense region to take into account disease progression. In cases where several days have passed after MRI imaging, the rate of progression of myelomalacia could not be determined accurately. In dogs, musculocutaneous nerves originate from C_7_ and C_8_, the radial nerve from C_8_ and the median and ulnar nerves from C_8_ and T_1_ [30]. Therefore, in cases operated more than 24 hours after MRI, the cranial extent of operation was set to T3 in cases with paresis of the thoracic limbs and set to T1 in cases with plegia of the thoracic limbs. Some dogs had abnormal macroscopic findings of swollen in the spinal cord at T3 or T1 during surgery, suggesting that intramedullary pressure was elevated due to progression of PMM. Accordingly, when swollen of the spinal cord at T3 or T1 was observed macroscopically after durotomy, an incision was extended to T2 or C7, respectively, of the anterior vertebral body. However, there is the possibility that this setting to cranial site of EHLD is too excessive. Further studies are necessary to evaluate minimal necessary extend of hemilaminectomy and durotomy.

One dog (No.23) which had tetraplegia died 3 days after surgery with respiratory failure. This finding suggests that cases with tetraplegia possibly have a higher risk of death after EHLD. According to the literature, the majority of dogs with presumptive and confirmed PMM are euthanized within 17 days of presentation [1]. Similar to a previous study [23], although EHLD was shown to be effective as a life-saving treatment for presumptive PMM in this study, there was no improvement of pelvic limb function. It is necessary to recognize that EHLD is not a surgery aimed at reversing plegia but at stopping the progression of myelomalacia. Pre-operative informed consent with extensive counseling is important for the owner to understand the role of EHLD in PMM and the long-term care required after surgery.

EHLD is highly invasive to the spine and spinal cord, therefore the risk of postoperative complications was also expected to be high. However, no serious complications caused by EHLD occurred in this study. Two dogs died from causes unrelated to the surgery approximately 2 months after EHLD. Importantly, these dogs did not have plegia of the thoracic limbs before surgery and no cases with plegia of the thoracic limbs died during follow-up. Although limitations of this study include its small sample size, forelimb plegia is unlikely to be a risk factor in long-term postoperative survival. However, in cases with residual thoracic limb plegia, there is a concern of a decrease in postoperative quality of life. Conversely, without thoracic limb plegia, walking with a wheelchair is possible and the owner’s satisfaction is likely to be higher.

There are several limitations in our study due to its retrospective nature. A definitive diagnosis of PMM requires post-mortem examination [1, 31]. Instead, the diagnosis of presumptive PMM in this study was based on clinical signs that reflected the progressive myelopathy. Additionally, all dogs had histopathologic confirmation of focal myelomalacia at the site of extruded intervertebral disc. However, the lack of postmortem confirmation of PMM does not allow us to eliminate the cases of focal myelomalacia. Previous studies suggested that the presence of a cerebrospinal fluid (CSF): L2_half − Fourier acquisition single−shot turbo spin−echo (HASTE)_ ≥ 7.4 times was higher sensitivity to diagnose presumptive and confirmed PMM than T2 hyperintensity used in the current study [1, 14]. Thus, CSF: L2_HASTE_ may use as a more reliable MRI criterion for the suspicion of PMM although this finding was not specific to PMM as well as T2 hyperintensity [1, 14]. A second limitation relates to the lack of a control group in the present study. It was because there were few cases followed up without EHLD in our hospital. Previous studies demonstrated a fatal course in dogs with presumptive and confirmed PMM [12, 23]. However, in almost all previous reports, the dogs described as PMM were not able to confirm a fatal progression because most dogs were humanely euthanased before respiratory failure results in their spontaneous death [1, 3, 20]. Moreover, some dogs diagnosed with presumptive PMM showed halting further progression after initial progression [20], which is similar to the findings obtained from human patients with subacute posttraumatic ascending myelopathy [32]. Thus, additional studies including control groups are necessary to address the presence of cases which did not have fatal progression even if untreated in our clinical setting.

## Conclusions

In summary, EHLD appears to be a life-saving treatment for PMM. Our algorithm of determining the range of EHLD is likely to be useful for setting the appropriate ranges of EHLD in the cases which develop signs consistent with PMM after MRI examination. However, the pathophysiology of PMM is unknown in many ways and further studies are required to develop new treatment methods.

## Methods

### Aim

To evaluate the effects of EHLD on halting the progression of PMM in dogs diagnosed with presumptive PMM which had a time lag between MRI and surgery.

### Design and setting

Retrospective review of 34 consecutive cases of presumptive PMM that have undergone EHLD at a private hospital.

### Case selection

The medical records of dogs diagnosed with IVDH at our referral and first opinion service from November 2014 through May 2020 were searched to identify cases with a presumptive diagnosis of PMM. According to the criteria of a previous study [1], a presumptive diagnosis of PMM was made based on neurological examination findings and the progression of clinical signs corroborated with MRI findings. All dogs presented with acute paraplegia and NDPP in both pelvic limbs and the tail. All dogs showed all the following additional clinical signs consistent with PMM: complete loss of pelvic limb reflexes, the loss of perineal and abdominal tones progressively, cranial advancement of the CTM reflex cut-off and a CTM reflex caudal border more than two vertebral levels cranial to the site of disc extrusion. All dogs had undergone MRI examination at multiple centres prior to surgery. MRI findings suggestive of PMM following IVDH were also taken into account as inclusion criteria. Extruded intervertebral disk material with spinal cord compression were detected by MRI in all dogs. The MRI findings of all dogs also showed intramedullary hyperintense region of the spinal cord > 6 times the length of L2 vertebral body on T2-weighted imaging [15]. Since extensive intramedullary T2 hyperintensity on MRI is not specific findings of PMM, dogs were excluded if the progression of the signs stopped prior to surgery. Dogs with NDPP in the thoracic limbs were also excluded. Informed consent for the EHLD procedure and information collection for research purposes were obtained. The review of MRI was performed using an Osirix 64-bit imaging software workstation (Pixmeo, Bernex, Switzerland) by the author (RH).

### Surgical procedure

The same anesthetic protocol was used for all dogs. Dogs were premedicated with 0.025 mg/kg atropine, subcutaneously. Anesthesia was induced using 0.5 mg/kg midazolam intravenously (IV), and Propofol (4 mg/ kg, IV, to effect). Anesthesia was maintained with isoflurane (1–2%) in 100% oxygen. Cefazolin sodium (25 mg/kg, IV) and 0.5 mg/kg morphine, were administered intramuscularly after induction.

**Fig. 3:**
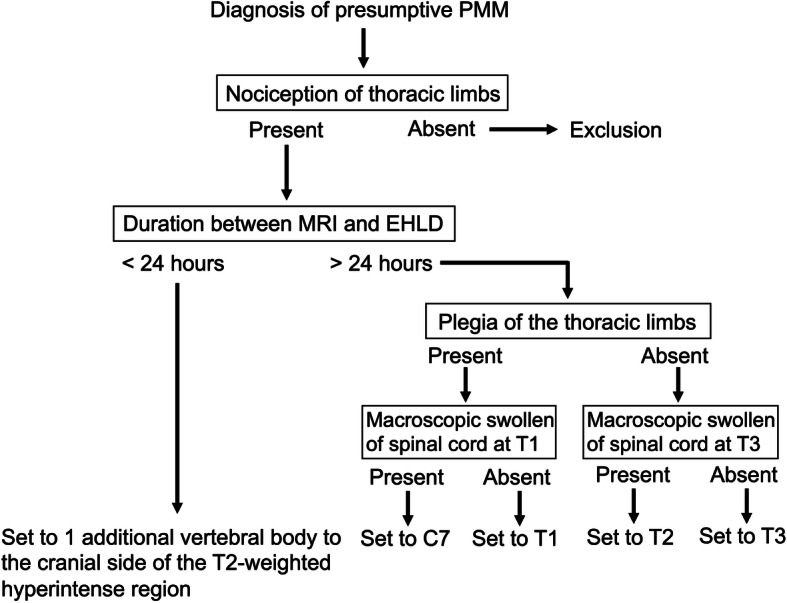
Algorithm to determine the level of the cranial site of the EHLD based on clinical features of dogs with presumptive PMM

Dogs were positioned in sternal recumbency. A dorsal approach was made to the T-L spine. At the beginning, hemilaminectomy was performed then the extruded disc material was removed. Subsequently, the dura mater of the spinal cord at the site was incised (Fig. 1a). After macroscopically verifying the presence of spinal softening and liquefaction at the IVDH site, EHLD was performed on the spinal cord (Fig. 1b). The hemilaminectomy and the durotomy were performed on the same vertebral body-length. In addition, the softened spinal cord at the site of extruded intervertebral disc was biopsied with a curette and fixed in formalin for histological diagnosis. A thin layer of subcutaneous fat graft was applied to the hemilaminectomy site before wound closure (Fig. 1c). The cranial side of the hemilaminectomy with durotomy range was set to 1 additional vertebral body to the cranial side of the T2-weighted hyperintense region as recognized by the MRI where the surgery was performed less than 24 hours after imaging (Fig. 3). In cases of more than 24 hours between MRI and surgery, the cranial site of the EHLD range was determined based on the neurological examination of the thoracic limbs and the macroscopic appearance during surgery (Fig. 3). The cranial site was set to T3 in cases with paresis of the thoracic limbs, and to T1 in those with plegia of the thoracic limbs (Fig. 3). If swollen of the spinal cord at T3 or T1 was seen macroscopically after durotomy, an incision was extended to T2 or C7, respectively, of the anterior vertebral body (Fig. 3).

### Perioperative care

Constant rate infusion of morphine (0.1–0.2 mg/kg/h, IV) was administered for the initial 48 hours after surgery. Prednisone (0.5 mg/kg, subcutaneously, 24 hours) was administered for 3–5 days after surgery. Cefazolin sodium (25 mg/kg, IV, every 12 hours) or Cephalexin (25 mg/kg, orally, every 12 hours) was administered for 10–14 days. Additionally, Chloramphenicol (25 mg/kg, IV, every 8 hours) was administered for 3–7 days to dogs with increased body temperature (> 39℃) [33]. Dogs were discharged from the hospital 10–21 days after surgery.

### Postoperative follow-up

Dogs were re-examined 2–4 weeks after discharge, and monthly re-examinations were conducted by the authors for 3–6 months to evaluate general health and neurologic signs. Dogs were also re-examined with radiographic evaluation of right lateral and dorsoventral views 2 weeks and 8 weeks after surgery by the author (RH) to evaluate a spinal luxation or subluxation. Subsequently, annual or biannual follow-up was performed by re-examination or telephone consultations.

## Data Availability

The datasets used and/or analysed during the current study are available from the corresponding author on reasonable request.

## Abbreviations

CP: Conscious proprioception
CSF: Cerebrospinal fluid
CTM: Cutaneous trunci muscle
NDPP: No deep pain perception
EHLD: Extensive hemilaminectomy with durotom
HASTE: Half-Fourier acquisition single-shot turbo spin-echo
IV: Intravenously
IVDH: Thoracolumbar intervertebral disk herniation
MRI: Magnetic resonance imaging
PMM: Progressive myelomalacia
